# Mental disorders in Malaysia: an increase in lifetime prevalence

**DOI:** 10.1192/bji.2021.4

**Published:** 2021-11

**Authors:** Shaeraine Raaj, Sujesha Navanathan, Myelone Tharmaselan, John Lally

**Affiliations:** 1 Specialist registrar in liaison psychiatry and neuropsychiatry, Department of Liaison Psychiatry, Mater Misericordiae University Hospital, Dublin, Ireland. Email: shaeraine@hotmail.com; 2 Department of Psychiatry, Royal College of Surgeons in Dublin, Ireland; 3 Ministry of Health Malaysia, Malacca, Malaysia; 4 Department of Psychiatry, Mater Misericordiae University Hospital, Dublin, Ireland

**Keywords:** Malaysia, mental health service, stigma and discrimination, Malaysian Mental Health Act 2001, mental healthcare delivery challenges

## Abstract

There is an increasing prevalence of mental disorders in Malaysia, with a growing need to improve access to timely and efficient mental healthcare to address this burden. This review outlines the current legislative framework and the challenges of delivering mental healthcare and treating mental disorders in Malaysia.

Situated in Southeast Asia, Malaysia is a melting pot of cultures. The population increased from 28.5 million in 2010 to 32.6 million in 2020, with 25.3 million people living in urban areas and 7.3 million residing in rural areas.^1^

Malaysia is known as an upper-middle-income country and the majority of the population are indigenous Malay (69.3%), with the remaining people either of Chinese (22.8%) or Indian (6.9%) ethnicity; minority ethnic groups represent 1% of the population.^1^ The median age of people in Malaysia is 30.3 years, with an average life expectancy of 73.2 years.^1^

Several policy reforms in the past decade have led to advances in the Malaysian mental health system. From the initial development of the Lunatic Ordinance of Sabah 1951 to the more recently implemented Mental Health Act 2001, there has been clear legislative, policy and organisational development to improve Malaysia's mental health services delivery. However, there remain many obstacles in the delivery of affordable and accessible mental healthcare.

## The burden of mental disorders in Malaysia

The most recent epidemiological data, published in 2015 by the Malaysian Ministry of Health, identified that the prevalence of mental disorders among adults was 29% (95% CI 27.9–30.5).^2,3^ This is a threefold increase in comparison with the 10% prevalence rate identified in 1996.^2,3^ The rural region of East Malaysia had the highest prevalence of mental disorders, at 43%, followed by the capital Kuala Lumpur, where 40% of the population fulfilled the criteria for a mental disorder.^2^ Rural regions have more adverse socioeconomic conditions, with higher poverty and unemployment. This, combined with increased stigma, reduced access to general and mental healthcare, and the practice of seeking alternative care through religious practitioners or shamans, can all contribute to an increased risk for the development and maintenance of mental health problems.^3,4,5^

There has been a dramatic increase in the prevalence of mental disorders over the past decade in Malaysia.^2^ Malaysia is transitioning from a middle-income country to a high income country, with rapid cultural and lifestyle changes due to increased urbanisation and globalisation, and associated increased levels of perceived stress. In the context of cultural changes, many are still affected by sustained economic difficulties, which contribute to social problems such as increased marital separation, changes in traditional parenting styles and the structure of family units, and an increase in alcohol and drug use. Further, increasing awareness of mental health problems by the public and by clinicians has likely led to increased reporting and identification of mental disorders. The National Health and Morbidity Survey of 2017 reported that the prevalence of suicidal ideation during the previous 12 months among adolescents in Malaysia was 10.0% (95% CI 9.2–10.8) and 6.9% (95% CI 6.2–7.7) of adolescents had attempted suicide one or more times during the previous 12 months.^6^ These findings suggest a fivefold increase in the prevalence of suicidal ideation among adolescents compared with 2011, when only 1.7% reported suicidal ideation.^6^ Moreover, the survey identified that suicidal behaviour was found to be highest among 13-year-old students, 10% of whom reported suicidal ideation, 9.0% had a suicidal plan and 10% had made a suicide attempt in the previous 12 months.^6^ These findings may suggest that 13-year-old students find the transition from primary school to secondary school stressful.^7^

Mental illness is one of the leading causes of disability and health loss in Malaysia, accounting for 8.6% of total disability-adjusted life-years (DALYs).^3^ The increasing prevalence of mental disorders in Malaysia is associated with an increased economic burden, with an economic analysis finding that mental health problems in the workplace were estimated to cost the Malaysian economy malaysian ringgit1 4.46 billion (£2.67 billion) in 2018.^3^

## Mental health legislation

The Malaysian Mental Health Act was passed in August 2001 by the Parliament in Malaysia and was implemented in 2010, following the Mental Health Regulation 2010.^8^ The provisions of the legislation include admission, detention, assessment, treatment and protection of a person with a mental illness.^8^

A person suspected of being mentally ill may be admitted involuntarily on application to the medical director of an acute psychiatry unit by a relative, police officer or social welfare officer following a personal examination no more than 5 days before the admission.^8^ The Malaysian Mental Health Act 2001 states that an involuntary patient can be discharged at any time by the medical director and does not specify a provision for an independent review of the detention.^8^ A Mental Health Tribunal provides a legal framework to safeguard individuals subject to compulsory treatment under national legislative and international human rights standards. The absence of a tribunal process places the Malaysian Mental Health Act outside of the United Nations universal right to exercise legal capacity identified in Article 12 of the UN Convention on the Rights of Persons with Disabilities. Limited attention was given to the legal obligation mandated under the United Nation's international human rights law while drafting the Malaysian Mental Health Act 2001.

## Access to treatment

A study in 2018 reported that Malaysia had a significant deficit of psychiatrists and psychologists, with a ratio of 1.27 psychiatrists per 100 000 population.^4^ There were 410 registered psychiatrists working in private universities, private clinics, public universities and government hospitals.^4^ The proportion of psychiatrists in Malaysia is higher than in other Southeast Asian countries such as the Philippines, with 0.52 psychiatrists per 100 000 population,^9^ but lower than in neighbouring Singapore, with 3.48 psychiatrists per 100 000 population.^4^ The capital of Malaysia, Wilayah Persekutuan Kuala Lumpur, has the highest ratio of psychiatrists, at 5.24 per 100 000 population, followed by Putrajaya at 3.38 per 100 000 population.^4^ Malaysia's rural states have the lowest number of psychiatrists, with 0.55 per 100 000 people in Kedah and 0.54 per 100 000 people in Sabah.^4^ Rapid urbanisation contributes to the uneven geographical distribution of doctors, with a decrease in numbers of psychiatrists in rural areas.^4^ There is a need for a uniform distribution of psychiatrists between all geographical locations within Malaysia, and Malaysia needs at least 3000 registered psychiatrists to meet the World Health Organization (WHO) recommendation of 10 psychiatrists per 100 000 population.^4^

The current model of care is divided into in-patient and community care. The primary care model is community-based, with 22 established community-based specialised mental health services (MENTARI) and 958 mental health day centres.^10^ Additionally, Malaysia has four mental health hospitals and 47 psychiatric in-patient units attached to general hospitals.^10^ Malaysia has used a more pragmatic approach to establish 38 in-patient units designated for children and adolescents.^10^

## Postgraduate psychiatry training

The Ministry of Health and the Conjoint Board monitors postgraduate psychiatry training in Malaysia.^4^ There are two parallel training pathways in operation: a postgraduate Masters programme in psychiatry and remote access to the Royal College of Psychiatrists’ (in London) Membership programme. The postgraduate training body provides a 4-year psychiatry Masters programme that involves completion of a three-part examination before the conferment of MMed (Psychiatry).^4^ In 2005 only three universities were providing the postgraduate Masters training programme in psychiatry.^11^ This has increased to six universities providing the programme in 2020. Since 2010, the parallel pathway has encouraged candidates to train for the Member of the Royal College of Psychiatrists (MRCPsych) qualification.^4^ It is prudent for the Ministry of Health to review the number of training posts allocated yearly and devise a plan to increase trainee psychiatrists’ numbers to meet the WHO recommendation.

Registered medical doctors in Malaysia must obtain 20 continuing professional development (CPD) points yearly to maintain their annual practice registration (APC) with the Malaysian Medical Council. As it stands, health agencies and non-governmental organisations (NGOs) are directed to deliver mental health conferences and seminars. These organisations are governed by the Ministry of Health and the Malaysian Medical Association.

## Challenges in delivering mental healthcare in Malaysia

The WHO Mental Health Atlas 2017 reported that upper-middle-income countries were found to spend a median of 2.4% of their health budget on mental health; however, in 2017 and 2018 respectively, the Ministry of Health had allocated 1.3% of the total health budget for mental health.^3,10^ As a result, it is a challenge for mental health services in Malaysia to sustain the current model of care, let alone to develop and deliver evidence-based practice.

A better understanding of stigma and of misunderstandings regarding mental illness can improve awareness and treatment. Lack of awareness and misconceptions about mental illness are a primary challenge in having access to treatment.^3,5,8^ It is estimated that only 20% of Malaysians with a mental disorder will access professional care, with social stigma a major explanatory factor for this. Owing to a lack of education about and understanding of mental illness, a cohort of traditionally conservative people in Malaysia tends to avoid medical treatment and seek religious practitioners or shamans.^5^ A study by Phang and colleagues reported that 54% of Malaysia's psychiatric patients had at least one contact with a traditional healer before engaging with psychiatric services.^5^ Previous studies identified that 62–69% first contacted a conventional healer for mental health problems.^5^

Indigenous ideas and culture lead to diverse interpretations and meanings of mental illness, with well-established local psychological theories and approaches embedded in the Malay culture.^5^ For Malaysians, the term psychiatric illness means *Gila* (crazy or madness), which carries a highly negative connotation. The majority of the Malay and Indian populations in Malaysia believe that mental health problems derive from spirit possession or social punishment.^5^ The Chinese cultural belief seeks to explain mental illness as being caused by a lack of spirit or the weakness of ‘Yin and Yang’, as well as problems related to self-worth, which is measured by material achievement, including education, occupation and monetary gain that brings the expected honour to the family.^5^ The three primary ethnic groups in Malaysia have their traditional healers: *vaidya* for the Indians, *bomoh* for the Malays and *sinseh* for the Chinese.^5^

Psychiatrists in Malaysia have incorporated psychospiritual therapy into clinical practice. This is a form of therapy designed to enhance the psychological well-being of people that was proposed by Risale-I Nur.^12^ Courage, love, hope, truthfulness, solidarity and sincerity are the core values in the psychospiritual dimension, and these are integrated into therapy.^12^ Rather than disregarding cultural beliefs about mental illness, it is necessary to continue engaging with communities to reduce stigma, increase education regarding mental illness and signpost where appropriate mental healthcare can be accessed.^5,12^

As mentioned above, the National Health and Morbidity Survey of 2017 reported that suicidal behaviour was found to be highest among students age 13.^6^ This may provide an opportunity for a collaboration between the Malaysian Ministry of Health and the Ministry of Education in terms of planning and delivering mental health awareness programmes to primary and secondary school students and teachers. The Great Minds Campaign was launched in 2019, targeting children, youth and members of the public. The mental health awareness campaign focused on three main areas, namely raising awareness, educating society about early warning signs of mental health issues and engaging with stakeholders. There is a need to design mental health awareness campaigns primarily focusing on school-aged children and adolescents.

## Conclusions

Overall, there have been significant reforms to mental health legislation in Malaysia, with service-level transition from custodial care to community care, and the development of a foundation of research and organisational development to improve mental health service delivery. Epidemiological studies are required to better understand causative factors for the increased prevalence of mental disorders in Malaysia, and to understand the contribution to this of emerging social changes in the context of increasing urbanisation. Broader policy changes may be required to incorporate consideration of the mental health impact of continued cultural and social development to promote security, education and social safety nets to enhance mental health. Social stigma and lack of awareness about mental health problems remain significant barriers to improving mental healthcare, and national mental health education programmes are required to address this. Increased mental health spending provision will be needed to address deficiencies in service availability and delivery and to increase the proportion of psychiatrists and mental health clinicians in Malaysia.

## Data Availability

Data availability is not applicable to this article as no new data were created or analysed in this study.

## Supplementary material

For supplementary material accompanying this paper visit https://doi.org/10.1192/bji.2021.4. click here to view supplementary material
