# Supplementary material for: Mental disorders in Malaysia: an increase in lifetime prevalence
Source: BJPsych Int. 2021 Nov;18(4):97–9. doi: 10.1192/bji.2021.4 (PMC8554924; doi:10.1192/bji.2021.4)
Supplement: Supplementary file 1 [file S2056474021000040sup.zip › S2056474021000040sup002.pdf]

Mental health in Malaysia: An increase in Lifetime Prevalence

Journal:	*BJPsych International*
Manuscript ID	BJPsychInt-20-0033
Manuscript Type:	Country Profile
Date Submitted by the Author:	27-May-2020
Complete List of Authors:	Raaj, Shaeraine; Phoenix Care Centre, Psychiatry Intensive Care Unit; RCSI; UCD<br>Navanathan, Sujesha; RCSI, Royal College of Surgeons in Ireland, Psychiatry; National Drug Treatment Centre, Addiction Services, Dublin, Ireland<br>Tharmaselan, Myelone; Ministry of Health Malaysia; Malaysian Medical Association<br>Lally, John; Institute of Psychiatry Psychology and Neuroscience, Psychosis Studies; Royal College of Surgeons in Ireland, Psychiatry
Keywords:	malaysian mental health service, Stigma and Discrimination, malaysian mental health act 2001, MENTAL HEALTH LEGISLATION, Challenges in delivering mental healthcare in Malaysia
Publishing Category:	Mental Health Services
Abstract:	Several policy reforms in the past decades have led to advances in the Malaysian mental health system, but translating this into reliable and effective mental health care remains challenging. From the initial development of the Lunatic Ordinance of Sabah 1951 to the more recently implemented Mental Health Act 2001, there has been clear legislative, policy and organizational development to improve delivery of mental health services in Malaysia. There is an increasing prevalence of mental disorders in Malaysia, with an increased need to improve access to timely and efficient mental health care to address this burden. This review outlines the challenges of delivering mental health care and treating mental disorders in Malaysia.

SCHOLARONE™
Manuscripts

Mental health in Malaysia: An increase in Lifetime Prevalence

Shaeraine Raaj 1-3, Sujesha Navanathan 2,4, Myelone Tharmaselan 5,6, John Lally[2,7,8]

1. Psychiatry Intensive Care Unit, Phoenix Care Centre, Dublin, Ireland;

2. Department of Psychiatry, Royal College of Surgeons in Dublin, Ireland;

3. Department of Psychiatry, School of Medicine, University College Dublin;

4. National Drug Treatment Centre, Addiction Services, Dublin, Ireland:

5. Ministry of Health Malaysia

6. Malaysian Medical Association

7. Department of Psychosis Studies, Institute of Psychiatry, Psychology and Neuroscience, King's College London, London, UK

8. St Vincent's Hospital Fairview, Dublin, Ireland

Abstract:

Several policy reforms in the past decades have led to advances in the Malaysian mental health system, but translating this into reliable and effective mental health care remains challenging. From the initial development of the Lunatic Ordinance of Sabah 1951 to the more recently implemented Mental Health Act 2001, there has been clear legislative, policy and organizational development to improve delivery of mental health services in Malaysia. There is an increasing prevalence of mental disorders in Malaysia, with an increased need to improve access to timely and efficient mental health care to address this burden. This review outlines the challenges of delivering mental health care and treating mental disorders in Malaysia.

For Peer Review

Malaysia, in Southeast Asia, is a melting pot of cultures with a population of over 32.6 million people (1). The population increased from 28.5 million people in 2010 to 32.6 million people in 2020 (1).

The majority (69.3%) of the population are Malays, with the remaining people either Chinese (22.8%) or Indian (6.9%) ethnicity (1). Minor ethnic groups represent 1% of the population.(1). Bahasa Malaysia is the national language of Malaysia, and English, the second language, is widely spoken .

Malaysia is a federation comprising of 13 states and 3 federal territories (1). Eleven states and 2 federal territories are located on the Malay Peninsula, called Peninsular Malaysia. The other 2 states and one federal territory are collectively called East Malaysia, and are also known as Sabah, Sarawak or Malaysian Borneo.

For Peer Review

Burden of mental disorders in Malaysia

The most recent epidemiological data reported by the Malaysian Ministry of Health (MOH) identified that the prevalence of mental health disorder among adults was 29% (95% CI: 27.9, 30.5) (2). The rural region of East Malaysia had the highest prevalence of mental health disorders at 43%, followed by the capital Kuala Lumpur, with 40% of the population meeting criteria for a mental health disorder (2).

The National Health and Morbidity Survey 2017 reported that prevalence of suicidal ideation during the past 12 months among adolescents in Malaysia was 10.0% (CI: 9.2, 10.8) and 6.9% (CI: 6.2, 7.7) of adolescents had attempted suicide one or more times during the past 12 months (3). These findings suggest a fivefold increase in the prevalence of suicidal ideation among adolescents in Malaysia compared to the year 2011 where only 1.7% people had suicidal ideation (3). More over, the survey identified that suicidal behaviour was found to be highest among form 1 students, where 10% had suicidal ideation, 9.0% had a suicidal plan and 10% had a suicide attempt over a 12 month period (3).

A recent study reported that mental health conditions in the workplace are estimated to cost the Malaysian economy RM14.46 billion (2.67 billion pounds) in 2018 (5).

MENTAL HEALTH LEGISLATION

The Malaysian Mental Health Act was passed on September 6, 2001, by the Parliament in Malaysia (6) and was implemented in 2010, following the enforcement of the Mental Health Regulations 2010 (6). The Act provided a structured framework for the comprehensive care of those with mental disorders and provided legislation for the admission, detention, assessment, treatment, and protection of a person with a mental disorder (6).

The Act describes mental disorder as any mental illness, incomplete development of the mind, or disability of the mind however acquired, in addition a person cannot be construed as suffering from a mental disorder by reason only of antisocial personality, sexual deviancy, or by intoxication of alcohol/drugs (6). A person suspected to be mentally ill may be admitted involuntarily upon application to the medical director by a relative, police officer or social welfare officer on a personal examination no more than 5 days before the admission of a person(6). The Malaysian Mental Health Act 2001 states that an involuntary patient can be discharged at any time by the Medical Director and does not specify a provision for tribunal(6).

For Peer Review

Service Gap

According to the World Health Organization (WHO), Malaysia has a significant deficit of psychiatrists and psychologists. A recent cross sectional study in 2018 reported that Malaysia has 410 registered psychiatrists working in private universities, private clinics, public universities and government hospitals, given a ratio of psychiatrist per 100,000 population in Malaysia of 1.27 (8). The number of psychiatrists is higher than other Western Pacific Rim countries such as the Philippines with 0.52 psychiatrists per 100,000 inhabitants (7), but lower than neighbouring Singapore with 4.4 psychiatrists per 100,000 populations (8, 11).The capital of Malaysia Wilayah Persekutuan Kuala

Lumpur has the highest ratio of psychiatrist of 5.24 per 100,000 populations followed by Putrajaya 3.38 per 100,000 populations (8). The rural states of Malaysia has the lowest number of psychiatrists, with 0.55 per 100,000 population in Kedah and 0.54 per 100,000 population in Sabah (8).

Postgraduate Training

The Ministry of Health (MOH) and Department of Postgraduate Committee (DPGC) is accountable for the postgraduate training in Malaysia.The postgraduate training body offers a four year psychiatric master program that include passing a three part examination before the conferment of M. Med (Psychiatry). In 2005 there were only three universities providing postgraduate masters training program in Psychiatry (9), and this has increased to six local universities in Malaysia providing these 4 year Masters program in Psychiatry as of 2020. There remains a need for the Ministry of Health to review the number of training posts allocated yearly and to devise a plan to increase the number of trainee psychiatrist to meet the WHO recommendation.

Registered medical doctors in Malaysia are required to obtain 20 Continuing Professional Development (CPD) points yearly to maintain their annual practise registration (APC) with the Malaysian Medical Council. The Ministry of Health (MOH) have a duty to organise and deliver mental health related conferences and seminars. These often have a particular focus on the learning needs of General Practitioners who provide first line care and need to identify mental illness in patients.

Challenges in delivering mental healthcare in Malaysia:

The current model of care is divided into inpatient and community care. The primary model of care is community based with 22 established community based specialised Mental Health Services (MENTARI) and a total of 958 mental health day centers (10). Additionally, Malaysia has 4 Mental Health Hospitals and 47 psychiatric inpatient units attached to a General Hospital (10). Malaysia has utilized a more pragmatic approach in establishing a total of 38 inpatient units designated for

For Peer Review

children and adolescents (10). The WHO Mental Health Atlas 2011 reports that upper middle income countries were found to spend a median of 2.4% of their health budget on mental health. Malaysia is classified as an upper middle income country, however in 2017 and 2018 respectively the Ministry of Health Malaysia had allocated 1.3% of the total health budget for mental health (10). In relation to this an increase on the mental health budget to 2.4% of the total health budget by the Ministry of Health (MOH) would bring Malaysia in line with the expenditure of the other middle-income countries.

A better understanding of stigma and misunderstanding regarding mental illness can contribute to improved awareness and treatment of psychiatric disorder. Lack of awareness and misconception about mental health is a primary challenge in having access to treatment (4,6). Due to a lack of education about and awareness of mental illness, a cohort of people in Malaysia tends to intentionally avoid medical treatment and seek religious practitioners or shamans (4). A study by Phang and colleagues reported that 54% of psychiatric patients in Malaysia had at least one contact with a traditional healer prior to first engagement with psychiatric services, with previous studies identifying that 62-69% first contacted a traditional healer for mental health problems (4).

Indigenous ideas and culture lead to diverse interpretation and meaning of mental illness, with well established local psychological theories and approaches embedded in the Malay culture . For Malays the term psychiatric illness means “Gila” (crazy or madness) which carries a highly negative connotation. The majority of the Malay society in Malaysia believes that mental health problems derive from spirit possession or social punishment. Besides that, the Chinese cultural belief seeks to explain mental illness as being caused by a lack of spirit or the weakness of “Yin and Yang” as well as problems related to self-worth, which is measured by material achievement including education, occupation and monetary gain that brings the expected honour to the family .

There is a need for a coordinated public mental health approach with comprehensive education, care and treatment available for people in local communities, and within existing structures.

For Peer Review

Opportunities to engage with people with mental illness at the point of contact must be made. This is highlighted by the finding that suicidal behaviour was found to be highest among form 1 students (3), which may provide an opportunity for a collaborative effort between the Ministry of Health (MOH) and Ministry of Education (MOE) in terms of planning and delivering mental health awareness programs to primary and secondary school students. There is a need to identify strategies to encourage and allow ease of access to care. A positive action that may be of sustenance in tackling the schooling cohort would include an increase in the teacher to student ratio, and a smaller classroom to enable a personable approach for students and teachers.

Rather than disregarding cultural beliefs regarding mental illness, it is necessary to engage with communities, reducing mental illness stigma, increasing education regarding mental illness and signposting where appropriate mental health care can be accessed. There remains untapped potential in local communities to integrate mental health services, either new or existing into existing community based support networks. Interventions can be modeled to the needs of local communities , be culturally adapted, while implementing evidenced based clinical practice when needed.

For Peer Review

Conclusion:

As it stands the Ministry of Health Malaysia has allocated a budget for curative, promotional and preventive work based on mental health. Community based specialised mental health services (MENTARI) were launched in 2015 across the country with progressive development. There are multiple factors which contribute to the increasing prevalence of untreated mental illness in Malaysia. The Ministry of Health (MOH) and policy makers need to address this crucial issue and develop preventive strategies to improve the mental well-being of Malaysians.

Conflict of interest. None.

Reference:

1. Department of Statistics Malaysia, 2019. Demographic statistics fourth quarter 2019, Malaysia.
2. National Health and Morbidity Survey (NHMS) 2015, Ministry of health Malaysia: Volume II: Non-Communicable Diseases, Risk Factors & Other Health Problems
3. National Health and Morbidity Survey (NHMS) 2017: Adolescent Health Survey 2017, Malaysia. Non-Communicable Diseases, Risk Factors & Other Health Problems.
4. Phang CK, Marhani M, Salina AA. Help-seeking pathways for in-patients with first-episode psychosis in hospital Kuala Lumpur. Malays J Med Health Sci. 2011;7:37–44.
5. Ning C. The economic cost of mental disorders in Malaysia .The Lancet Psychiatry, April 2020. Volume 7, Issue 4, e23
6. Khan NN, Yahya B, Abu Bakar AK, Ho RC. et al. Malaysian mental health law. BJPsych international. 2015;12(2):40-42
7. Lally, J., Tully, J., & Samaniego, R. (2019). Mental health services in the Philippines. *BJPsych international*, *16*(3), 62–64. https://doi.org/10.1192/bji.2018.34
8. Guan, N.C., et al., Psychiatrists in Malaysia: The ratio and distribution. Malaysian journal of psychiatry,2018. 27(1).
9. Deva MP. Psychiatry and mental health in Malaysia. Int Psychiatry. 2005;2(8):14-16. Published 2005 Apr 1.
10. World Health Organization WHO's Mental Health Atlas 2017. Country Profile. Malaysia
11. Singapore Medical Council. Annual report 2018.

For Peer Review
